# From Theory to Measurement: Recommended State MCH Life Course Indicators

**DOI:** 10.1007/s10995-015-1767-1

**Published:** 2015-06-30

**Authors:** Tegan Callahan, Caroline Stampfel, Andria Cornell, Hafsatou Diop, Debora Barnes-Josiah, Debra Kane, Sarah Mccracken, Patricia McKane, Ghasi Phillips, Katherine Theall, Cheri Pies, William Sappenfield

**Affiliations:** Field Services Office, Office for State, Tribal, Local and Territorial Support, Centers for Disease Control and Prevention, Dekalb County, GA USA; Association of Maternal and Child Health Programs, Washington, DC USA; Office of Data Translation, Bureau of Family Health and Nutrition, Massachusetts Department of Public Health, Boston, MA USA; Lifespan Health Services Unit, Nebraska Department of Health and Human Services, Lincoln, NE USA; Field Support Branch, Division of Reproductive Health, National Center for Chronic Disease Prevention and Health Promotion, Centers for Disease Control and Prevention, Dekalb County, GA USA; Bureau of Family Health, Division of Health Promotion and Chronic Disease Prevention, Iowa Department of Public Health, Des Moines, IA USA; Women’s and Children’s Health Section, Division of Public Health, North Carolina Department of Health and Human Services, Raleigh, NC USA; Maternal Child Health Epidemiology Unit, Lifecourse Epidemiology and Genomics Division, Michigan Department of Community Health, Lansing, MI USA; Division of Community Health Promotion, Florida Department of Health, Tallahassee, FL USA; Department of Global Community Health and Behavioral Sciences, Tulane University School of Public Health and Tropical Medicine, New Orleans, LA USA; School of Public Health, University of California, Berkeley, CA USA; College of Public Health, University of South Florida, Tampa, FL USA

**Keywords:** Health indicators, Life course, Public health surveillance, Maternal and child health, Reproductive health

## Abstract

**Purpose:**

In May 2012, the Association of Maternal and Child Health (MCH) Programs initiated a project to develop indicators for use at a state or community level to assess, monitor, and evaluate the application of life course principles to public health.

**Description:**

Using a developmental framework established by a national expert panel, teams of program leaders, epidemiologists, and academicians from seven states proposed indicators for initial consideration. More than 400 indicators were initially proposed, 102 were selected for full assessment and review, and 59 were selected for final recommendation as Maternal and Child Health (MCH) life course indicators.

**Assessment:**

Each indicator was assessed on five core features of a life course approach: equity, resource realignment, impact, intergenerational wellness, and life course evidence. Indicators were also assessed on three data criteria: quality, availability, and simplicity.

**Conclusion:**

These indicators represent a major step toward the translation of the life course perspective from theory to application. MCH programs implementing program and policy changes guided by the life course framework can use these initial measures to assess and influence their approaches.

## Significance

Although current public health surveillance systems provide data that can be used to assess life course health components, this is the first multistate consensus on indicators to define and monitor life course health at the state level.

## Introduction

The life course approach to maternal and child health (MCH) includes the full spectrum of factors that influence an individual’s health through all stages of life. The life course approach to MCH is grounded in life course theory. Life course theory first emerged in the fields of sociology and developmental psychology in the early 1900s and resulted in appeals for longitudinal approaches to research.^1^ Later health researchers began to observe the relationship between early life experiences and subsequent health outcomes; particularly pioneering for life course theory within health research was the work on fetal origins of adult health.^2^ Research informed by life course theory was applied early on in MCH to racial disparities in birth outcomes^3^ and has evolved over time into a lifecourse health development model which defines health through understanding dynamic, emergent processes and interactions between risk and protective influences throughout the lifespan.^4^

In recent years, corresponding with—and in response to—the development of a lifecourse health development model, there has been expanding interest in life course approaches to public health practice among health departments and community partners across states and within communities.^5^^,^^6^ As an operational concept for MCH public health practice, life course theory has been used as a framework explaining the relationship between health trends and disparities by focusing on the biological, social, economic, and environmental factors underlying population health experiences and outcomes.^7^ As more stakeholders examine health through a life course lens, assessment and evaluation tools are required to help assess risk and resilience factors; quantify and illustrate the connected community structure needed to support a life course approach to public health; and aid in the planning of comprehensive, integrated systems and programs.

Currently, there are no nationally standardized population-based metrics for measuring a life course approach to MCH. In response, the Association of Maternal and Child Health Programs (AMCHP), an association of state health department Title V MCH programs, launched a project designed to identify and recommend a set of state-level life course indicators that can be used to assess, monitor, evaluate, and advocate for programs and policies for MCH populations. This article describes the multistate collaborative methodology used to develop the proposed indicators, presents a list of indicators selected from currently available national surveys and data systems, and explores the strengths and limitations of the selected indicators.

## Methods

### Organizing Framework

Throughout early 2012, 25 national thought leaders from academia and public health practice were convened as part of the Life Course Metrics National Expert Panel. The panel developed an operational definition for “life course approach” for the overall project, recommended a four-part framework to use in proposing indicators, and suggested initial criteria for the screening and evaluation of possible indicators.

As defined by the national expert panel:

A life course approach is based on a theoretical model that takes into consideration the full spectrum of factors that impact an individual’s health, not just at one stage of life (e.g., adolescence), but through all stages of life (e.g., infancy, childhood, adolescence, childbearing age, elderly age). Life course theory shines light on health and disease patterns—particularly health disparities—across populations and over time. Life course theory also points to broad family, social, economic, and environmental factors as underlying causes of persistent inequalities in health for a wide range of diseases and conditions across population groups.

Table 1 contains core components of a life course approach.

**Table 1:** Core components of a life course approach

A life course approach is a stages of life theory that takes into consideration factors that impact an individual’s health and development through all stages of life, from preconception health into infancy, and through childhood, adolescence, and childbearing years into older age
This approach considers the influence of family, environmental, biological, economic, behavioral, social, and psychological impacts on health outcomes across the lifespan
Critical or sensitive periods of development in early life can affect exposures and experiences; this impact may influence health and disease patterns and outcomes later in life
These influences may have potential cumulative effects on health outcomes (i.e., health at any given stage of life is a function of experiences at prior stages), and one cannot understand adult health without addressing child health
Health promotion and prevention interventions can be directed toward different stages of life
Connections exist between life stages (e.g., the relationship between adolescence and the two life stages that border it: childhood and adulthood)
Efforts should be coordinated both across life stages and across the life span

Based on this definition, a four-part framework was recommended to help states think broadly about potential indicators that move beyond traditional performance measures. The national expert panel envisioned a set of indicators that captured the role of MCH programs across four areas: minimizing risk, improving outcomes, providing services, and maintaining or expanding capacity. Translated to a framework, these four elements are defined as (1) Risks—the experiences and exposures that indicate risk for future life course outcomes; (2) Outcomes—the health and social outcomes that reflect or summarize an adverse life course trajectory; (3) Services—the risk reduction and health promotion from services provided over time to MCH populations; and (4) Capacity—the capacity of communities and organizations to address health through a life course perspective.

**Fig. 1:**
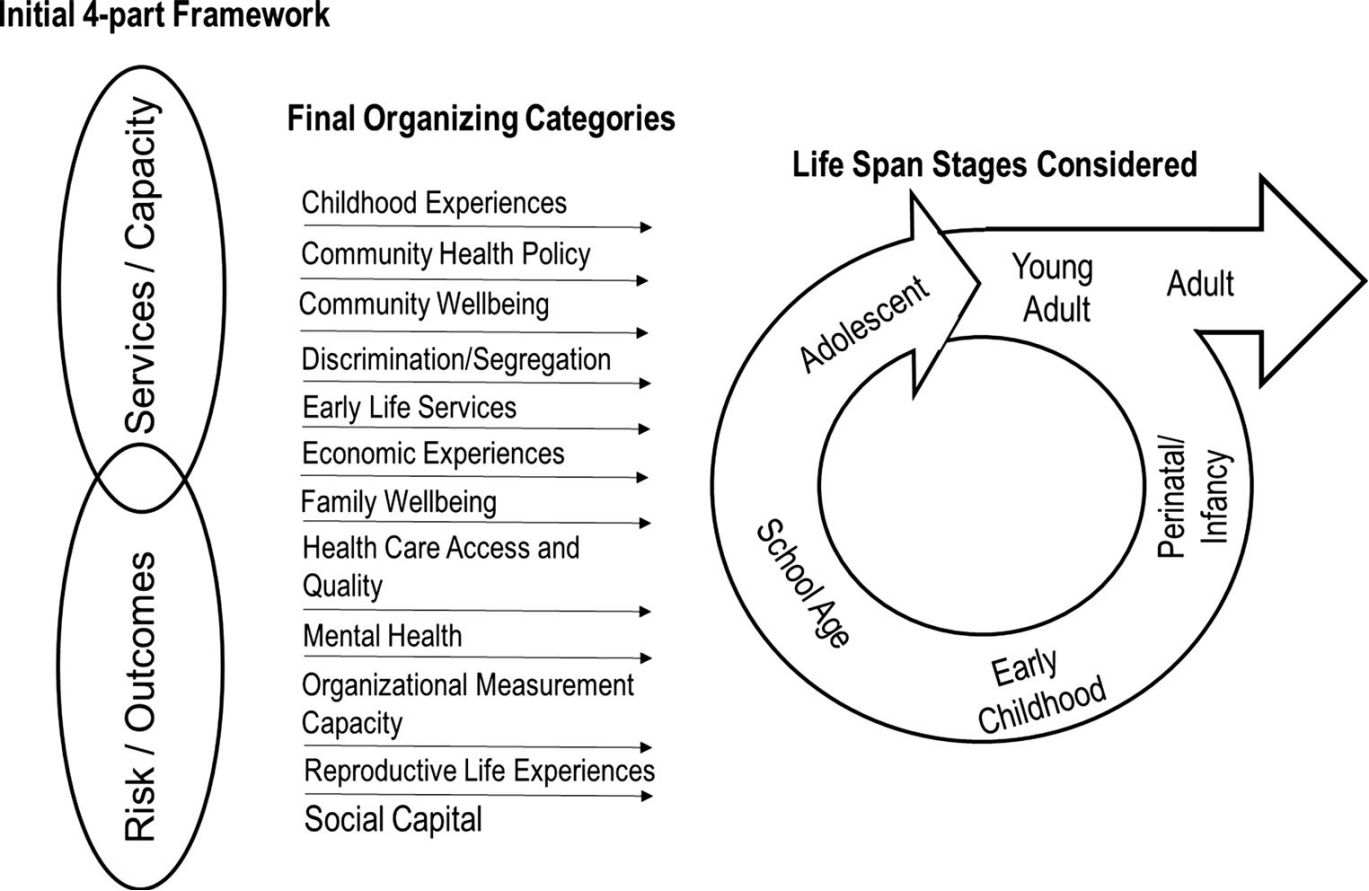
Evolution of the organizing framework for life course indicators

The initial four-part framework created challenges because of overlap in the concepts that define risk and outcome indicators, as well as the concepts that define services and capacity indicators. During indicator selection, the initial four-part framework was condensed into two overarching categories: Risk/Outcome and Capacity/Services. In condensing the four-part framework to two large categories, the teams still needed a pragmatic way to organize the final 59 indicators. To meet this need, the indicators were assigned to 12 categories that describe the scope and diversity captured in the set while avoiding disease- or population-specific identifiers. The evolution of our organization framework is represented in Fig. 1.

### State Teams

Life course theory is an extensive, complex, and multifaceted approach, and identification of life course indicators was therefore best served through a collaborative, multiorganizational effort engaging state teams inclusive of experts from state public health programs, state epidemiology and data programs, community health and social service providers, public health academics, and other cross-sector partners. Seven state-based teams were selected to lead indicator selection through a competitive process in which applicant teams were asked to describe current commitment to a life course approach to MCH and describe a working team that represents the multidimensional aspects of life course. In August 2012, the selected teams began the process of developing and rating state MCH life course indicators. After discussions with the national expert panel, the state teams finalized the organizing framework. The teams used the framework as a platform to generate indicator proposals and develop the final set of criteria to rate the indicators.

### Indicator Criteria

Criteria were established to help screen, evaluate, and determine the strength of potential life course indicators. Each indicator was assessed on five core features of a life course approach: equity, resource realignment, impact, intergenerational wellness, and life course evidence. Indicators were also assessed on three data criteria: quality, availability, and simplicity. Expanded definitions of these criteria are included in Table 2.

**Table 2:** Descriptions of indicator criteria used throughout screening and selection

Criterion	Description of criterion
A life course approach—core features	
1. Equity	The indicator reflects and has implications for equity-related measures such as social, psychosocial, and environmental conditions, poverty, disparities, and racism
2. Resource alignment	Health and illness are influenced by multiple interacting factors from many different contexts such as social, psychosocial, and environmental conditions. The indicator is reflective of programs, services, and policies that expand beyond the traditional MCH focus
3. Impact	The public health impact of a positive (increase or decrease depending on the indicator) change in the indicator due to program or policy interventions
4. Intergenerational wellness	The indicator reflects the time and trajectory components of the life course theory with an emphasis on indicators that address critical and transitional periods throughout life
5. Life course evidence	The indicator is connected to our current, scientific understanding of life course health
Data—core features	
1. Availability	The data for this indicator available in each of the public health agencies in the 50 states and the District of Columbia
2. Quality	Quality data is available for measuring the indicator
3. Simplicity	The indicator is simple to calculate; and easy to explain the meaning and use of indicator to professionals and the public

The five core features criteria were also used to evaluate how well each selected indicator incorporates components of a life course approach to MCH and to argue for why it should be considered an appropriate life course indicator. For example, infant mortality, though a sentinel indicator of the health of populations, was not included in the final set of indicators. When considering the life course criteria, state teams decided an appropriate life course indicator would illuminate the risk and protective factors that influence infant mortality and affect child development. The final set of indicators does include important risk and interim outcome components of infant mortality, such as preterm birth, small for gestational age, maternal education, experiences of discrimination, and economic measures.

### Indicator Selection

AMCHP facilitated an eight-step process to support state teams in selecting the recommended indicators. State teams review the expert panel’s work and the proposed selection process and approved with minor modifications. The process was implemented with small modifications based on previous experiences with public health indicator selection, including the preconception health indicators and the chronic disease indicators.^8^^,^^9^The process is outlined below.

1. *Call for indicator proposals.* The state team and national expert panel members issued a call for proposals. In addition, a call was issued to the general public and publicized on the AMCHP website, through AMCHP publications, and through partner networks.
2. *Initial screen of indicator proposals.* Via email, members of the seven state teams rated each indicator based on how well it met the defined criteria. During a 2-day, in-person meeting, representatives from the state teams voted “yes/no” on further consideration of each proposed indicator. To make it onto the initial list of selected indicators, each indicator had to be approved by supermajority—at least five of the seven team representative votes.
3. *Development of indicator description sheets.* State team members or AMCHP staff constructed a description for each indicator selected in the initial screening round.
4. *Final screen and vote on indicator proposals.* After reviewing and considering indicator description sheets, state teams provided overall ratings of indicators. State team representatives met for a second, in-person meeting to discuss the indicator proposals and select a final set of indicators through vote of the supermajority.
5. *Release of final indicator selections for public comment.* Public comment was solicited through a variety of channels, including listserves, targeted emails, webinars, and special presentations to interested groups. State and local health departments, federal agency representatives, state and national nonprofit organizations, and a number of interested individuals submitted comments.
6. *Refinement of final indicators based on feedback.* The indicator set was refined based on feedback from the public comment period. Proposals to drop or replace particular indicators and to make changes to numerator, denominator, or data source were made. Each proposal was presented to the state teams for consideration, and modifications were made accordingly. Ultimately, no indicators were added or dropped. The comments were used to refine indicator definitions and to develop and strengthen information supporting each indicator in the final set.
7. *Dissemination of final indicator set.* After revisions were made based on public comment, the final indicator set was disseminated through the AMCHP website. An online indicator tool provides indicator information, including the expanded indicator description sheets where numerator, denominator, possible modifiers, national comparison data (when available), and notes on calculation are summarized alongside descriptions of how the indicator meets the data and life course criteria.
8. *Development of tools for use.* In addition to the online indicator tool mentioned above, tools were developed to promote the use of the indicators and make them accessible to a variety of stakeholders.

## Results

State teams, national expert panel members, and the public submitted proposals for 413 indicators using the organizing framework. The first round of rating, discussing, and voting resulted in the selection of 102 indicators for consideration through research and development of indicator description sheets.

After the final round, the teams recommended a final set of 59 indicators. Recommended indicators are drawn from 28 separate data sources, with 40 of the 59 indicators drawn from eight data sources (summarized in Table 3) and twenty indicators drawing from a unique data source., The 59 final indicators were organized into the 12 descriptive categories: 3—Childhood Experiences; 2—Community Health Policy; 6—Community Wellbeing; 5—Discrimination and Segregation; 3—Early Life Services; 3—Economic Experiences; 11—Family Wellbeing; 8—Health Care Access and Quality; 4—Mental Health; 3—Organizational Measurement Capacity; 8—Reproductive Life Experiences; and 3—Social Capital.

**Table 3:** List of the most common data systems used in the life course indicator selection and development process (sources for two or more indicators)

Data system source	Description	Number of indicators from source^a^
Behavioral Risk Factor Surveillance System (BRFSS)	Telephone health survey tracking health conditions and risk behaviors. Administered by the US Centers for Disease Control and Prevention since 1984 in partnership with state and local programs. Currently, data are collected monthly in all 50 states, the District of Columbia, Puerto Rico, the US Virgin Islands, and Guam for adults 18 years and older	8
National Survey of Children’s Health	A survey sponsored by the Maternal and Child Health Bureau of the Health Resources and Services Administration, which examines the physical and emotional health of children aged 0–17 years. The survey is administered using the State and Local Area Integrated Telephone Survey methodology, and it is sampled and conducted so that state-level estimates can be obtained for the 50 states, the District of Columbia, and the Virgin Islands	7
National Vital Statistics System	An intergovernmental sharing of data whose relationships, standards, and procedures form the mechanism by which the National Center for Health Statistics (NCHS) collects and disseminates the nation’s official vital statistics. Vital event data are collected and maintained by the jurisdictions that have legal responsibility for registering vital events; these entities provide the data via contracts to NCHS. Vital events include births, deaths, marriages, divorces, and fetal deaths. In the United States, legal authority for the registration of these events resides individually with the 50 states, 2 cities (Washington, DC, and New York City), and 5 territories (Puerto Rico, the Virgin Islands, Guam, American Samoa, and the Commonwealth of the Northern Mariana Islands)	7
Pregnancy Risk Assessment Monitoring System (PRAMS)	An ongoing population-based surveillance system designed to identify and monitor selected maternal experiences and behaviors that occur before and during pregnancy and during the child’s early infancy. It is administered by CDC in partnership with forty states and New York City, representing approximately 78 % of all US live births	5
Youth Risk Behavior Surveillance System (YRBSS)	Includes a national school-based survey conducted by CDC; state, territorial, and local education and health agencies; and tribal governments. The YRBSS monitors priority health-risk behaviors and the prevalence of obesity and asthma among youth and young adults	5
American Community Survey	An ongoing nationwide survey that collects and provides annually data on demographic, social, economic, and housing in the United States. The survey is administered by the US Census Bureau and, starting in 2010, replaced the decennial census long form	4
National Survey on Drug Use and Health (NSDUH)	Administered annually by the Substance Abuse and Mental Health Services Administration, the NSDUH measures the prevalence of use of illicit drugs, alcohol, and tobacco in the civilian, noninstitutionalized US population aged 12 years old or older. Data collection was conducted periodically 1971–1990 and has been conducted annually since 1990. The survey uses a combination of computer-assisted personal interviewing to obtain basic demographic information, and audio computer-assisted self-interviewing for most of the questions	2
National Immunization Survey(NIS)	A list-assisted random-digit-dialing telephone survey followed by a mailed survey to children’s immunization providers to monitor childhood immunization coverage. The study, conducted by CDC, collects data by interviewing households in all 50 states, the District of Columbia, and selected large urban areas. The target population for the NIS is children between the ages of 19 and 35 months living in the United States at the time of the interview. Estimates are produced for the nation and geographic areas consisting of the 50 states, the District of Columbia, and selected large urban areas. Data files for the NIS are available starting with 1995	2

^a^Note the total N will not sum to 59 as some indicators have components from multiple data sources

The predominant reasons why potential indicators were excluded during the selection process were the following: indicator data were frequently not available at a state level for the majority of US states and the District of Columbia; indicator sensitivity, specificity, positive predictive value, reliability, and consistency across jurisdictions were not of the desired quality; or the indicator was too complex to calculate and/or explain to professionals and the public when balanced with the value gained from its calculation. In addition, state teams considered duplication or similarity of indicator focus and alignment with current life course science. A list of potential indicators that were not selected was made available to inform stakeholders of the scope of indicators considered and to advocate for the development of surveys and data systems to address identified gaps.

Critical issues emerged when applying the life course criteria, in particular two of the criteria—implications for equity and impact across the lifespan. State team members opted to use a broad definition of equity that did not focus solely on racial and ethnic differences, and they adopted the perspective that any population disparity in a risk factor or health outcome should be viewed as an inequity. A number of indicators were initially proposed as being important across the lifespan, but further discussion revealed that for each of these indicators, one or more critical and sensitive life stages had the most impact for a person’s life trajectory. State teams were asked to examine global indicators critically to determine whether they should be revised to focus on the most critical/sensitive life stages.

Table 4 provides a brief description of the set of 59 recommended life course indicators, organized by descriptive category. Project resources, including an online tool that provides in-depth information about each indicator, were released in the fall of 2013 and are available on the AMCHP website.^10^

**Table 4:** Descriptive category, indicator name and brief description

ID	Category	Name and/or brief description
LC-1	Childhood experiences	Prevalence of adverse childhood experiences among adults
LC-2	Childhood experiences	Prevalence of adverse childhood experiences among children
LC-3	Childhood experiences	Substantiated child maltreatment including experience of physical abuse, neglect or deprivation of necessities, medical neglect, sexual abuse, psychological or emotional maltreatment
LC-4	Community health policy	Breastfeeding support—Baby-Friendly Hospitals: proportion of births occurring in baby-friendly hospitals
LC-5	Community health policy	Fluoridation: proportion of population served by community water systems that received optimally fluoridated water
LC-6	Community wellbeing	Concentrated disadvantage: proportion of households with high level of concentrated disadvantage, calculated using 5 census variables
LC-7A	Community wellbeing	Homelessness: prevalence of homelessness among individuals
LC-7B	Community wellbeing	Homelessness: prevalence of homelessness among families
LC-8	Community wellbeing	Homicide rate: homicides per 100,000 population
LC-9	Community wellbeing	Household food insecurity
LC-10	Community wellbeing	Poverty: percentage of population living under the Federal Poverty Level (FPL)
LC-11	Community wellbeing	Small for gestational age: proportion of singleton live-born infants whose birth weight is at or below the 10th percentile for a given gestational age
LC-12	Discrimination and segregation	Bullying: percentage of 9th–12th graders who reported being bullied on school property or electronically bullied
LC-13	Discrimination and segregation	Experiences of race-based discrimination or racism among women: percentage of women who experienced discrimination right before or during pregnancy
LC-14	Discrimination and segregation	Perceived experiences of discrimination among children: percentage of children who experienced discrimination in the past year (parent report)
LC-15	Discrimination and segregation	Perceived experiences of racial discrimination in health care among adults
LC-16	Discrimination and segregation	Racial residential segregation, by community: differential distribution of individuals by race or other social or income factors (Dissimilarity Index)
LC-17	Early life services	Early intervention: proportion of children aged 0–3 years who received early intervention services compared to all children aged 0–3 years
LC-18	Early life services	WIC nutrition services: proportion of children aged 2–5 years receiving WIC services compared to proportion of children <185 % FPL
LC-19	Early life services	Early childhood health screening—Early periodic screening, diagnosis and treatment: percentage of Medicaid-enrolled children who received at least one initial or periodic screen in past calendar year
LC-20	Economic experiences	High school graduation rate: high school graduation rate (4-year cohort) as measured by the Adjusted Cohort Graduation Rate
LC-21	Economic experiences	Mother’s education level at birth: percentage of births by maternal education levels
LC-22	Economic experiences	Unemployment: prevalence of unemployment
LC-23	Family wellbeing	Adolescent smoking: percentage of adolescents who smoked cigarettes in the past 30 days
LC-24	Family wellbeing	Adolescent use of alcohol: percentage of adolescents using alcohol during the past 30 days
LC-25	Family wellbeing	Children with special healthcare needs: percentage of children (0–17 years) with special healthcare needs
LC-26	Family wellbeing	Diabetes: percentage of adults with diagnosed diabetes
LC-27	Family wellbeing	Exclusive breastfeeding at 3 Months: percentage of children exclusively breastfed through 3 months
LC-28	Family wellbeing	Exposure to secondhand smoke in the home: percentage of children living in a household where smoking occurs inside home
LC-29	Family wellbeing	Hypertension: percentage of adults with diagnosed hypertension
LC-30	Family wellbeing	Illicit drug use: prevalence of illicit drug use in the past month among population aged 12 years or older
LC-31	Family wellbeing	Intimate partner violence, injury, physical or sexual abuse: number of intimate partner victimizations per 1000 persons aged 12 years or older
LC-32A	Family wellbeing	Childhood obesity: percentage of children who are currently overweight or obese
LC-32B	Family wellbeing	Adult obesity: percentage of adults who are currently overweight or obese
LC-33	Family wellbeing	Physical activity among high school students: proportion of high school students who are physically active for at least 60 min per day on five or more of the past 7 days
LC-34	Health care access and quality	Cervical Cancer Screening: proportion of women who receive the appropriate evidence-based clinical preventive services (Pap smear) for cervical cancer screening
LC-35	Health care access and quality	Children receiving age-appropriate immunizations: percentage of children aged 19–35 months receiving age-appropriate immunizations according to the Advisory Committee for Immunization Practices guidelines and Healthy People 2020 goal
LC-36A	Health care access and quality	Human papillomavirus (HPV) immunization: proportion of adolescents aged 13–17 years who receive the evidence-based clinical preventive service HPV vaccine
LC-36B	Health care access and quality	Human papillomavirus (HPV) immunization: proportion of young adults aged 18–26 years who receive the evidence-based clinical preventive service HPV vaccine
LC-37	Health care access and quality	Medical home for children: proportion of families who report their child received services in a medical home
LC-38	Health care access and quality	Asthma emergency department utilization: proportion of persons on Medicaid with asthma having an asthma emergency department visit
LC-39	Health care access and quality	Inability or delay in obtaining necessary medical care, dental care, or prescription medicines: percentage of parents reporting their child was not able to obtain necessary medical care or dental care
LC-40	Health care access and quality	Medical insurance for adults: proportion of adults with medical insurance
LC-41	Health care access and quality	Oral health preventive visit for children: percentage of children who received a preventive dental visit in the past 12 months
LC-42	Mental health	Depression among youth: percentage of 9th–12th graders who felt sad or hopeless almost every day for more than 2 weeks during the previous 12 months
LC-43	Mental health	Mental health among adults: percentage of adults with poor mental health
LC-44	Mental health	Postpartum depression: percentage of women who have recently given birth who reported experiencing postpartum depression following a live birth
LC-45	Mental health	Suicide: suicides per 100,000 population
LC-46	Organizational measurement capacity	Capacity to assess lead exposure
LC-47	Organizational measurement capacity	Data capacity to support integrated childhood research: ability of state MCH programs to support integrated, population-based childhood research (i.e., research using linked program data). For state level, proportion of priority datasets to which the MCH program always has timely access (including for linkage) for program or policy planning purposes. For national level, proportion of states that have timely access to at least 5 priority datasets
LC-48	Organizational measurement capacity	States with P-20 Longitudinal Data Sets: states with P-20 W longitudinal data systems. A P-20 W is a data system in which policies and standards are aligned to link student data for specified purposes across the education continuum, from early childhood through K-12, postsecondary education, and the workforce
LC-49	Reproductive life experiences	Diabetes during pregnancy: percentage of adult women with diagnosed diabetes during pregnancy only
LC-50	Reproductive life experiences	Early sexual intercourse: initiation of sexual intercourse before age 13 years
LC-51	Reproductive life experiences	HIV prevalence: HIV rate per 100,000 total population
LC-52	Reproductive life experiences	Postpartum contraception: proportion of women using birth control postpartum
LC-53	Reproductive life experiences	Repeat teen birth: percentage of teen births that are repeat teen births
LC-54	Reproductive life experiences	Teen births: number of live births per 1000 females aged 10–19 years
LC-55	Reproductive life experiences	Preterm birth: percentage of live births born <37 weeks gestation
LC-56	Reproductive life experiences	Stressors during pregnancy: proportion of women reporting two or more stressors during pregnancy
LC-57	Social capital	4th Grade proficiency: percentage of 4th graders scoring “proficient” or above on math and reading
LC-58A	Social capital	Incarceration rate: prevalence of juveniles aged 13–17 years, male or female, detained in residential placement
LC-58B	Social capital	Incarceration rate: prevalence of adults incarcerated
LC-59	Social capital	Voter registration

## Discussion

Within the final indicator set, there is overlap with existing public health measures. This overlap demonstrates the synergy of a life course approach with other public health approaches and programs. Furthermore, this agreement across initiatives illustrates how the reframing of MCH through a life course approach does not require starting from scratch. Rather, the data that are already collected are integrated and can provide a starting point into this new framing of MCH to identify opportunities for investment in and applications of life course. The overlap also provides a helpful opportunity for engaging with new partners who may not be familiar with life course by identifying the life course components of current initiatives. Sixteen of the recommended indicators are current Title V performance measures,^11^ 8 are core state preconception health indicators,^12^ 36 align with federal Healthy People topic areas and objectives,^13^ 14 are national Chronic Disease Indicators,^14^ 6 align with the Center for Disease Control and Prevention’s Winnable Battles initiative,^15^ and 9 are measures endorsed by the National Quality Forum.^16^

In addition to the existing measures used in MCH, there are indicators not as commonly used for MCH programs. Examples include fluoridation, concentrated disadvantage, homelessness, perceived experiences of discrimination, racial residential segregation, organizational data measurement capacity, and voter registration. These indicators help expand the focus of MCH programs to incorporate broader economic and social opportunities, community capacity and policy, and the living and working conditions experienced by individuals.

The indicator criteria favored the selection of an indicator set that builds bridges among partners to articulate a shared vision and promotes novel approaches to building capacity, improving services, and reducing exposure to risk factors. The final indicator set has the ability to help MCH programs leverage new and existing partnerships through the inclusion of nontraditional MCH indicators. Using these indicators to define assessment and evaluation of a life course approach to MCH will require considering a breadth of investments and partners influencing health. To achieve measurable change within any of these indicators, multi-sector partnerships among agencies at the federal, state, and local levels, as well as schools, urban planners, community- and faith-based organizations, national-to-local initiatives, and more must work together within a collective impact framework.^17^

Despite the strengths of the final indicator set, there are also limitations based on current data availability. Specifically, the lack of indicators measuring resiliency and protective factors. Although life course theory includes resiliency factors in addition to risk factors, current public health practice is primarily focused on risk measurement. From an epidemiology perspective, tracking disease prevalence and mortality has been the prevailing public health approach; most standard measures in epidemiology tend to be risk-based. True resiliency measures, however, are not necessarily the opposite of risk measures. Further work is needed to identify factors that truly support or counterbalance risks in the life course approach to MCH. Despite this challenge, the recommended indicator set offers a few examples of resiliency measures, including Fourth Grade Proficiency (LC-57), Voter Registration (LC-59), and multiple measures of receipt of immunizations and/or preventive care.

Another major weakness is the lack of indicators based on longitudinal data. The operating assumption for selecting the indicators was that they could be used immediately when released. The current availability of data at the state and local levels limited what could be considered as indicators; the lack of readily available longitudinal data is one example of how this restriction creates gaps in the set. Potential longitudinal indicators include having measures that examine the combination of various risk and/or resilience factors. Lastly, the complexity of some of the proposed indicators posed a challenge for the simplicity criteria. A proposed indicator may have truly captured the life course implications for how an economic factor influences health, but if it was so complex to calculate and explain that no one could easily use or understand it, it was not considered an appropriate life course indicator.

With an overall lack of available, longitudinal data within state public health data systems the final indicator set is also limited by the cross-sectional nature of the indicators included. Critical developmental periods is a key aspect of life course science and the life course development model.^18^^,^^19^^,^^20^ The cross-section indicators within the final set, therefore, cannot be specifically tied back to related critical periods as defined through research on life course development. While this is a limitation for the operationalization of the final set, the accompanying indicator description available for each indicator on the AMCHP website provides more thorough discussion for each indicator on the relation of the cross-section measure to critical developmental periods and processes.

## Conclusion

Life course theory provides a rich and layered understanding of the development of an individual’s health over time and across generations. The theory emphasizes the role of timeline, timing, risks, resiliency, environment, and equity on individual health.^21^ The components of life course theory require public health practitioners to emphasize the linking and integration of programs; promote integrated multi-sector service systems; ensure the availability of services at critical and sensitive periods throughout the lifespan; incorporate whole person, whole family, and whole community approaches into all work; and address health equity through working toward elimination of health disparities.^22^ Several state and local MCH programs and initiatives are using the life course theory to form priorities and develop plans for public health programs. Participants from the multistate collaborative are beginning to use the indicators and resources to help align initiatives with a life course approach, broaden their collaborations through engagement of new stakeholders, leverage new partnerships, and develop data-to-action plans. Specific examples are summarized in Table 5. Although current public health surveillance systems provide data that can be used to assess life course health components, this is the first multistate consensus on indicators to define and monitor life course health at the state level.

**Table 5:** State approaches to using the MCH life course indicators

State	Approaches and examples
Florida Department of Health	Provided funding to add questions to the 2014 Florida Behavioral Risk Factor Surveillance System (BRFSS) to gather more life course information for the state, including adverse childhood experiences, intimate partner violence, and perceived racial discrimination in health care
	Will use MCH life course indicator description sheets to inform needs assessments for the Title V and Title X competitive grant applications throughout 2014, including to broaden Florida’s current base of stakeholders and leverage partnerships for focus areas
	Plans to create a statewide Life Course Indicator Report to set benchmarks that will assist MCH programmatic efforts
Iowa Department of Public Health	Bureau of Family Health is incorporating MCH life course indicators into a larger evaluation framework, which includes the alignment of all MCH-related metrics across the life course according to the public health impact pyramid,^a^ including Title V Performance and Outcome Measures, Title X Family Planning indicators, newborn screening performance indicators, and other relevant MCH measures
	Will translate alignment to a framework to (1) evaluate program quality and gaps in programming, (2) design or enhance surveillance systems, and (3) draft or update policy
Massachusetts Department of Public Health	Will integrate MCH life course indicators into the Title V MCH Needs Assessment and new priorities/performance measures in 2015, in alignment with a priority selected in 2010 (“Promote continuity of care and Life Course Model with an emphasis on social determinants of health to improve coordination of services across all MDPH programs across the lifespan”)
	Will use indicators to shape state’s health improvement plan for state public health accreditation
	Included measures of racism and discrimination (measures included among the life course indicators) on the PRAMS survey in 2009 and 2010 and will likely continue collecting these data in the future
	WIC program will use a selection of the indicators when planning/updating a performance management initiative
Michigan Department of Community Health	Division of Family and Community Health has integrated the MCH life course indicators into a broader framework for tracking health across the life course to inform policymakers and stakeholders about the health status of Michigan residents and reinforce the concept that health status is integrated with and dependent on community, social determinants of health, and system capacity
	Will use analysis of the MCH life course indicators as an innovative way to describe a conceptual framework for integrating core outcomes across the stages of the MCH life course with core community capacity and system infrastructure indicators
	Will use indicators for strategic decision making, supporting improved collaboration, and identifying gaps in programming and opportunities for improvement
Louisiana Department of Health and Hospitals and Tulane University	The Bureau of Family Health and Tulane are in the process of linking some of the Economic Experiences, Discrimination and Segregation, and Social Capital and Community Engagement indicators to state and local data sets. Louisiana PRAMS data have been linked to segregation data from the US Census
	Working to geocode PRAMS and birth outcome data to examine and understand the influence of macro community factors on racial/ethnic and socioeconomic status disparities in MCH outcomes
	Updating existing data systems to better report on the MCH life course indicators: adverse Childhood Experiences items and measures of discrimination that have been included on the latest PRAMS survey will be included on the next BRFSS survey

^a^Frieden [22]

## Footnotes

1 Russ et al. [1].

2 Barker et al. [2].

3 Lu and Halfon [3].

4 Halfon et al. [4].

5 Frey et al. [5].

6 Shirmali et al. [6].

7 Fine and Kotelchuck [7].

8 Broussard et al. [8].

9 Centers for Disease Control and Prevention [9].

10 Association of Maternal and Child Health Programs [10].

11 Health Resources and Services Administration [11].

12 Broussard et al. [12].

13 US Department of Health and Human Services [13].

14 Centers for Disease Control and Prevention [14].

15 Centers for Disease Control and Prevention [15].

16 National Quality Forum [16].

17 Kania and Kramer [17].

18 Scott [18].

19 Power and Hertzman [19].

20 Hertzman et al. [20].

21 Fine and Kotelchuck [21].

22 Fine and Kotelchuck [21].
